# Defining Polyamory: A Thematic Analysis of Lay People’s Definitions

**DOI:** 10.1007/s10508-021-02002-y

**Published:** 2021-05-27

**Authors:** Daniel Cardoso, Patricia M. Pascoal, Francisco Hertel Maiochi

**Affiliations:** 1 Department of Sociology, Manchester Metropolitan University, Manchester, M15 6EB UK; 2 Escola de Comunicação, Artes e Tecnologias, Universidade Lusófona de Humanidades e Tecnologias, Lisboa, Portugal; 3 CICPSI, Faculdade de Psicologia, Universidade de Lisboa, Lisboa, Portugal; 4 Escola de Psicologia E Ciências da Vida, Universidade Lusófona de Humanidades E Tecnologias, Lisboa, Portugal; 5 CPUP, Faculdade de Psicologia e, Ciências da Educação da Universidade Do Porto, Porto, Portugal

**Keywords:** Polyamory, Laypeople, Thematic analysis, Consensual non-monogamy

## Abstract

This study aimed to analyze laypeople’s definitions of polyamory and compare definitions presented by people who are not willing to engage in consensual non-monogamy (CNM) and those who are or are willing to be in a CNM relationship. This exploratory qualitative study used data collected from a convenience sample through a web survey, where people answered the question “What does polyamory mean?” We conducted thematic analysis to examine patterns in meaning and used demographic data to compare themes among groups. The final sample comprised 463 participants aged 18–66 years (M = 32.19, SD = 10.02), mostly heterosexual (60%). Of the total sample, 54% were in a monogamous relationship, followed by 21% not in a relationship, and 13% in a non-monogamous relationship. Analysis showed that people define polyamory mostly as a set of behaviors in a relationship, followed by the potential of multiple relationships or feelings for multiple people. Definitions also include emotional, sexual, and ethical aspects. People in CNM relationships are more likely to define polyamory as constituting a potential form of relating, focus more on interpersonal feelings and ethics, and include consent in their definitions than those unwilling to engage in CNM. People in CNM relationships also focus particularly on the non-central role of sex within these relationships, which might challenge assumptions about sexuality in these relationships in clinical and research settings.

## Introduction

Polyamorous relationships have become more visible in the media since the term “polyamory” was coined in the early 1990s. The use of the term showed more visible growth compared to other terms describing types of consensual non-monogamy (CNM), such as swinging. This demonstrates growing public awareness about polyamory as compared to other forms of CNM (Cardoso, 2010, 2020; Moors, 2017). Research on the prevalence of polyamorous relationships is scarce; currently, there are estimates on the prevalence of specific forms of non-monogamy worldwide or in particular countries, but little concrete data. Sexually non-monogamous relationships are relatively common, with some data positing that over 21% of singles in the U.S. have been in a sexually non-exclusive relationship at some point in their lives, with no association with race, socioeconomic status or education, and positive associations with being male and with bisexuality or homosexuality (Haupert et al., 2017). A recent study measured the prevalence of polyamory in the U.S. and estimated a point prevalence of 0.6–5% and a lifetime prevalence of 2–23% (Rubel & Burleigh, 2020).

The term “polyamory” was created in two distinct contexts (Cardoso, 2010) within a two-year interval. It was first used by Morning Glory Zell-Ravenheart in a 1990 newsletter of the neo-pagan Church of All Worlds, inspired by the science fiction book *Stranger in a Strange Land* by Robert A. Heinlein. The second context is attributed to Jennifer Wesp, who was looking for a word that could serve as a synonym for “non-monogamy” and ended up naming her mailing list “alt. polyamory” in 1992 (Cardoso, 2010). Since then, several definitions have been suggested and debated. According to Klesse (2006), “polyamory is a contested term,” resisting clear definitions and being debated and questioned since its inception by multiple individuals and groups, with different objectives. Some of the main aspects of this contestation have to do with the role of sex in CNM (Klesse, 2005), whether it is necessarily politically engaged (Wilkinson, 2010), and whether it should focus more on individuals or communities (Cardoso, 2015).

Many definitions used in academia are derived from popular books on polyamory and non-monogamy and popular mailing lists and blogs that helped create the term and spread its use. The derivations were an attempt to align those definitions with the views and experiences of the people in these communities, thereby allowing those definitions to better reflect their lived experiences (Ritchie & Barker, 2006). Other sources include glossaries and dictionaries, whose entries were populated by activists such as the previously mentioned Morning Glory Zell-Ravenheart. The sourcing of definitions in academia can be seen, for instance, in Barker (2005, p. 75), where polyamory was defined as a “relationship orientation” based on a set of beliefs according to which it is possible and acceptable to “love many people and to maintain multiple intimate and sexual relationships”; further examples are based on popular books such as *The Ethical Slut* (Easton & Liszt, 1997) and *Polyamory: The New Love Without Limits* (Anapol, 1997). This pattern agrees with research on other identities whose direct naming does not historically stem from pathological conventions, even though they are surrounded by attempts at pathologization (e.g., asexuality; Alcaire, 2015).

Rubel and Burleigh (2020) used academic and specialized popular press sources to categorize different aspects of polyamory. In doing so, they noted that these sources mostly focus on polyamory as (1) a belief or preference, (2) a relationship status, or (3) a relationship agreement, with some including love and longevity as mandatory elements. The authors then created a survey disseminated through Amazon’s Mechanical Turk that measured the prevalence of polyamory when defined as one of the abovementioned three categories and a new category they named “identity.” Respondents were asked to define polyamory, and their responses were split into *basic* definitions (“if they mentioned multiple partners […] or mentioned being in love with multiple individuals, and if they did not imply that it necessarily involved marriage” [Rubel & Burleigh, 2020, p. 18]) and *comprehensive* definitions (when there was some mention of consent). The results of this study prove that, as mentioned above, definitions are fundamental for research, prevalence analysis changes considerably depending on the definition used, and simpler definitions of polyamory (i.e., those that do not include consent) are prevalent. However, these categories were derived using a top-down approach, which we seek to complement by not deploying an a priori definition of polyamory. We argue that our approach complements existing studies and definitions by allowing for a broader understanding of polyamory, one that is less bound by theoretical or academic preconceptions.

Existing definitions change on multiple levels, such as their characteristics and meanings for individuals, implications for identity, and management of the public perception of polyamory. Ritchie and Barker (2006) argued that in a social constructionist approach, “The language around us shapes our self-identities” and “our understanding of sexual identity depends on the language of sexuality available to us” (p. 585). Differences in definitions might translate into different possibilities or restrictions for identity and behavior. The existing multiple definitions—including those available for mainstream culture where CNM is often represented as cheating within the context of compulsory monogamy—are also contested by academics, activists, and polyamorous persons. This reflects a reduced vocabulary regarding possibilities of identities, feelings, and behaviors, validating only some identities (Conley et al., 2012, 2013).

There is evidence that polyamory, as well as other forms of CNM, suffer social stigma, being valued as less desirable or even harmful to people and society (Burris, 2013; Cardoso & Ribeiro, 2016; Conley et al., 2012, 2013; McCrosky, 2015; Rodrigues et al., 2018; Séguin, 2019). In this context, stigma has detrimental effects at various levels an in different contexts. One of these contexts is health care. For example, CNM is often associated with a supposed higher chance of contracting sexually transmitted infections, which research does not support (Conley et al., 2012, 2013). This stigma extends to mental health professionals, who have been shown to sometimes have a willingness to persuade clients to not engage in CNM relationships (Grunt-Mejer & Łyś, 2019), or hold mononormative bias when engaging with clients (Brown, 2015; Jordan, 2018; McCoy et al., 2015).

Polyamorous relationships are perceived as less committed, less trusting, and more likely to lead to disease (Conley et al., 2012, 2013; Séguin, 2019). These assertions are made despite evidence against them, as health and happiness levels reported by people in non-monogamous relationships are equal to or higher than those of people in monogamous relationships (Conley et al., 2012, 2013; Fleckenstein & Cox, 2015). However, stigma prevents proper health care from being delivered to polyamorous persons that could arguably suffer from minority stress (Meyer, 2003), as research seems to indicate (Cardoso et al., 2020), and this minority stress may be amplified by the expectation of encountering prejudice and rejection by social groups, or health care professionals (McCrosky, 2015).

Another context where stigma has detrimental effects is human rights. Societal perception and stigma also impact social and political rights (Cardoso, 2014) and their relation to a State-incentivized mononormativity (Klesse, 2016). CNM exists in a state of *alegality*—not illegal but also not legally recognized as valued or worthy of welfare considerations (Santos, 2019)—although this situation is slowly beginning to change in some places (Snyder & Alsharif, 2020). Non-monogamous marriage is often criminalized (Donoso, 2009; Hooper, 2014; Klesse, 2016).

Differing modes of defining polyamory, or other forms of CNM, have societal repercussions, as the intimate practices that are privileged, excluded, or reinforced impact individuals’ identities and societal perceptions of polyamory itself, including stigmatized perceptions. As such, when constructing possible definitions of polyamory, researchers should take potential stigma into consideration. That is to say, the existence of stigma—as reported by CNM individuals—should be factored into how definitions interact with stigma and its analysis, and how they serve as vehicles for negotiating stigma. One example is the study conducted by Kean (2018), who argues that many definitions of polyamory avoid the inclusion of sexual behavior or minimize its importance, making polyamory seem less about promiscuity; this minimizes stigma and makes the practice better received than others that have a more explicitly sexual focus, such as swinging.

Beyond the health and societal impacts of the conceptual definitions, these are also important to academic research on CNM relationships. To develop research, clear definitions are needed. Sometimes, research on polyamory employs definitions created by the researchers themselves or by educators and activists within polyamorous communities (Cardoso, 2010; Matsick et al., 2014). At other times, they end up using umbrella terms that include concepts and practices like swinging and open relationships, such as “consensual non-monogamy” (Haupert et al., 2017). “Consensual non-monogamy” still has monogamy as the focus, however, and this exact detail was one of the self-declared motivators for Jennifer Wesp to coin a neologism (Cardoso, 2010).

Although academic definitions of polyamory are mostly grounded in discussions by self-identified polyamorous people, they do not reflect how laypeople understand the term. In this study, we strive to understand how laypeople define polyamory without knowing any previous prompts or definitions. As other authors mention (Hogg & Williamson, 2001), defining “laypeople” is not straightforward, especially since there are many layers of what can constitute expertise (professional, biographical, technical aspects, among others). Here, we define “laypeople” as participants who were not specifically recruited from professional (health professionals or academics) or activist circles related to relationship styles or sexuality and who were not asked to pronounce themselves as experts or professionals. Looking at definitions from people’s day-to-day understandings will help inform scientific and clinical practice, as shown by research conducted on similar areas (Buck, 2016; Saunders et al., 2007).

From a social constructivist perspective, people’s experiences, identities, desires, and relationships are shaped by the culture they live in. Analysis focusing on laypeople’s understanding may provide insight on how the culture perceives polyamory and inform general and professional attitudes toward polyamorous people. This approach might also reveal distinctions in understanding among people according to their own connection to the topic, showing whether stigma might partially be derived from a (mis)understanding of polyamory. Finally, it might also show which definitions used in academia best represent laypeople’s perception of polyamory in the non-monogamous population in general. This meta-definitional work will help build more accurate scientific models based on empirically validated definitions and will help illuminate the difference between definitions of people who are in CNM relationships and of those who are in a monogamous relationship and would not consider the possibility of being in a CNM relationship. Moreover, the work will also encourage a better understanding of prevalent stereotypes or ideas regarding CNM relationships, which in turn might help educate the public on the same topics.

### Study Objectives

This study initially aimed to analyze the definitions of polyamory given by laypeople. This analysis would help health and education professionals and inform social advocacy and policy makers to better understand public perceptions of polyamory and adjust their practice accordingly; this would minimize stigma, potential prejudice, and miscommunication. Moreover, we aimed to inform further investigations on polyamory with definitions obtained from laypeople’s definitions. After data collection and in the light of intergroup relations and social identity theory, as a secondary goal, we sought to illuminate the thematic differences regarding the definition of polyamory among people currently in CNM relationships (in-group) and people currently in monogamous relationships who are not willing to be involved in CNM relationships (out-group).

## Method

### Participants

We gathered 609 responses initially. After removing 146 responses that were blank or unintelligible, 463 valid answers remained. In this sample, age varied between 18 and 66 years (M = 32.19, SD = 10.02). Most participants were heterosexual (60%, n = 278), followed by bisexuals (18%, n = 84), gay/lesbian (13%, n = 60), other (5%, n = 23), queer (2%, n = 9), and undefined (2%, n = 9). The most prevalent relationship status was “currently in a monogamous relationship” (54%, n = 250), followed by people currently not in a relationship (32%, n = 152) and people currently in a non-monogamous relationship (13%, n = 61). Over half of the participants had a college degree or higher education qualification (52%, n = 240), and most lived in urban areas (80%, n = 370).

As stated above, although there were no specific checks related to profession, based on data collected about education and the number of Portuguese-speaking researchers that we know are conducting research on CNM (around a dozen, mostly concentrated in the Lisbon area), we consider this sample goes beyond academia and activists and successfully provides a laypeople approach to the topic. Nonetheless, we note specific limitations to this point at the end of the paper.

### Procedure

The study received ethical and deontological approval by the CEDIC-Ethics and Deontology Committee for Scientific Research. The data analyzed in this study was gathered within the scope of a larger online preliminary study about CNM that used qualitative and quantitative data. In the current manuscript, we conducted a thematic analysis of responses to the question “How would you define polyamory?”.

The survey was presented in Portuguese. Data were collected from a convenience sample using a web survey form published on social media in a snowball-like method.

After reading the information about the study, participants were requested to provide consent to participate in the survey and gain access to it. The question was included in a larger survey that encompassed sociodemographic questions related to age, education levels, relationship status, sexual orientation, gender, and living context (rural or urban) as well as other questions and scales that are beyond the scope of the current manuscript; some of the other manuscripts have already been published (Cardoso et al., 2020). The open question was asked after the sociodemographic questions and before the measures were presented. Participants were instructed not to look online for definitions, as our goal was to understand the immediate definition people would think of without the aid of online sources. Since all of the data was collected in Portuguese and all researchers speak Portuguese as their main language, the complete analysis (including the development of themes, sub-themes, and codes, as detailed below) was conducted in Portuguese. This allowed us to preserve the specificity, detail, and cultural context of the responses for as much of the process as possible and allowed for parsimony when handling the data. After concluding the analysis, we translated the thematic map as well as the ad verbatim examples presented below into English.

To define the in- or out-group status, we followed the criteria based on social identity theory (Tajfel & Turner, 1979); according to the theory, in-groups are those that people identify with and out-groups are those that people do not identify with and may present stigma and discrimination against. We did not collect data that allowed us to determine the in- and out-group status based on self-categorization. As Becker (1997) noted, being targeted by discrimination or considered by others as part of a group does not necessarily involve belongingness or self-identification.

Nevertheless, informed by the study of Sizemore and Olmstead (2018), we considered that self-reporting unwillingness to be consensually involved in a non-monogamous relationship would be a reliable/valid indicator of out-group status. Therefore, in the monogamous group, we included those people who reported involvement in monogamous relationships and also expressed unwillingness to be involved in any form of CNM, as measured by Item 5 of the Willingness to Engage in Consensual Non-Monogamy Scale (Sizemore & Olmstead, 2017). All people involved in a monogamous relationship or who said they were not involved in any relationship and who expressed some degree of willingness to engage in CNM were excluded from our analysis, because we could not accurately evaluate the meaning of these people’s willingness to engage in CNM. Finally, we arrived at a split between people involved in CNM (in-group, n = 55) and those in monogamous relationships who are not willing to be involved in CNM (out-group, n = 157).

### Data Analysis

To find the answers to the aforementioned question, we used reflexive thematic analysis as described by Braun and Clarke (2013) and Braun et al. (2014). To analyze the responses collected, we applied inductive, semantic, and (critical) realist approaches to the thematic analysis. This means we used the explicit content of the responses to inform the development of themes. Each data point (i.e., each answer given) could be assigned multiple codes, depending on the density or amount of content in the answer. We followed the steps provided in Braun and Clarke’s (2006) method for the thematic analysis. First, all the responses were read multiple times, allowing the researchers to familiarize themselves with the data. After this, initial codes were generated. Codes represent the most basic unit in the raw data that can be analyzed meaningfully. We coded for semantic content that seemed relevant to the question at hand. We chose to privilege semantic content over latent content, as the size and depth of answers left too wide a margin for possible interpretations of latent content. As the average response size was relatively short, we chose to code whole answers as units for analysis, preserving the context of the response.

After the initial coding, all the codes were analyzed and gathered in sub-themes according to their topical proximity and conceptual similarity. Subsequently, the sub-themes were aggregated under main themes. These themes and sub-themes were the main objects of analysis and reflected patterns in the data set. The themes and sub-themes generated were then reviewed in their correspondence to the coded extracts that composed them and against the entire data set in an iterative process until a thematic map comprehensive of the data and showing how each theme related to the others was generated. Then, themes’ names were reviewed for clarity, as each theme had to be clear in what it did and did not represent. Finally, a report was written to show the study’s conclusions. To assure the validity of the research, the first and third authors coded the responses autonomously. The thematic maps were compared and checked until a consensus was reached and then presented to the second author, who analyzed the categories through a critical approach, leading to another review and then the creation of the final version; the same process has been followed in other recent studies (e.g., Pascoal et al., 2019; Santos et al., 2020).

We used QSR Nvivo 12 to import the data and assist in the coding and analysis process. After developing a consensual final thematic map (see Table 1), which aggregated the results from all the respondents, we proceeded to contrast definitions presented by people involved in CNM with those provided by people in a monogamous relationship who were not willing to engage in CNM.

**Table 1:** List of the themes, sub-themes, and codes (with ad verbatim examples) from the thematic analysis

Main themes	Sub-themes	Codes	Description	Examples Ad verbatim
Emotion Behavior	Intrapersonal	Loving	Polyamory is loving more than one person	Loving many people
		Attraction	Polyamory is being attracted to more than one person	Romantic attraction for more than one partner simultaneously
		Essentialism	Polyamory is natural or intrinsic to some people	Innate
	Interpersonal	Intimacy	The relationship is an intimate one	When the person is available for intimate relationships with many people
		Romance	The relationship is a romantic one	When a romantic relationship is maintained with more than one person
		Affection	The relationship is an affectionate one	A form of affectionate and/or sexual relationship
		Compersion	There are feelings of compersion or absence of jealousy	Consented love among many people, without jealousy
		Love	The relationship is a loving one	Loving relationship with many people
		Equality of feelings	People are supposed to feel equally about all their partners	Multiple relationships, with equal love
	Relating	Relationship	Polyamory is having relationships with people	Loving relationship with more than one partner
		Family and Cohabitation	Relationships that are about building families and living together	An open relationship among more than two people living together
		Longevity	How long relationships should last	Accepting the possibility of having long-lasting, loving, and intimate relationships
		Stability	Relationships are stable, serious, or committed	Many intimate, serious relationships
		Openness	Polyamorous relationship is open to new partners	Open relationships based on consent, knowledge, respect, and establishment of rules
		Structure	Concerns about relationship structure, and how different partners interact with each other	Having many loving partners who might or might not be involved with each other
	Ethics	Knowledge	All people involved know of each other	Loving many people at the same time with the knowledge of all of them
		Consent	All people involved consent to be in the relationship	Consensual loving relationships with many partners
		Respect and honesty	References about respecting partners and valuing honest communication and trust	Intimate relationships based on respect and consent
		Explicit frameworks	Mentions of specific frameworks, such as feminism or religion	A non-monogamous, ethical, feminist relationship format
		Rules	Mentions of rules and limitations within relationships	A relationship between at least three people with rules defined by all of them
	Sexuality	Relationship is sexual	Relationships that involve sex and physical contact	Loving and sexual relationship with more than one person
		Sex is optional	Relationships that aren’t necessarily sexual	Intimate relationship with many people, independent of sexual acts
Potential	From Within	Possibility	Polyamory is something that is possible even when not made concrete	Possibility of having multiple relationships simultaneously
		Capability	Having the capability for feelings or relationships	Capable of loving many people
	From Without	Ability to	Being able to develop feelings or pursue relationships	A person that can have multiple relationships, and their partners can also have many relationships"
		Freedom to	Having the freedom to feel or pursue relationships	Freedom to develop loving relationships

To compare these two groups, we sought to understand if there were notable differences in the thematic composition of the answers given when compared to the general thematic map created. This involved understanding whether there were any shifts in the centrality of different themes across the groups, whether any specific codes were absent in one of the groups, and whether the themes present were more positively or negatively connotated (e.g., self-evident use of sarcasm, derogatory language). Keeping in line with the method used, in spite of using NVivo 12, we did not resort to any form of quantitative analysis or comparison to facilitate the separation of responses into the two groups after the general coding.

## Results

As noted above, the initial objective of the study was to analyze laypeople’s definitions of polyamory in a way that could illuminate the best practices of both research and health care. Therefore, all responses were looked at *in toto*, and answers were not separated into any groups prior to coding.

The secondary analysis arose from an unexpected number of respondents in several different types of relationship configurations and differing degrees of willingness to engage in CNM. As differences found in the secondary analysis did not warrant another thematic map, a single one was considered to be the most parsimonious form of presenting the data.

Most answers were short and included the notion of a plural relationship or feeling for more than one person. Some answers elaborated these short definitions with different characteristics and some outlined conditions for the establishment of these relationships or feelings. A minority of answers were elaborate and had more complex terminology, such as compersion or queerplatonic relationships. Some participants made a point of using LGBTQ-inclusive language, and very few used academic and activist-connotated language. Some people employed examples with strict gender roles, and very few gave criticisms against polyamory, most notably stating they did not believe in polyamory as real love or they saw polyamory as a way to manipulate people into sex.

**Fig. 1:**
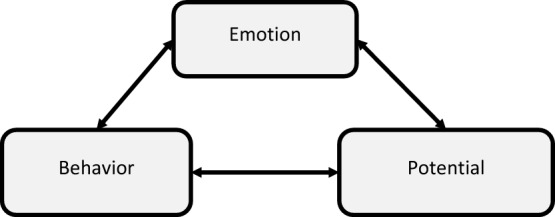
Thematic map of the answers to the research question “How would you define polyamory?”

The resulting codes were organized into sub-themes, which were then aggregated in main themes (see Table 1) that included *ad verbatim* examples for each code. As main themes, the final thematic map included Emotion, Behavior, and Potential, as presented in Fig. 1.^1^ Overall, this means that the responses mainly associate polyamory with emotions, behaviors, or potentials (whether extrinsic or intrinsic to individuals) or any combination of these three dimensions. Over the next few paragraphs, we explain how each theme and sub-theme can be better understood by looking at what was coded under them.

The sub-themes and codes were related and not mutually exclusive; for example, a definition could encompass codes from both emotion and behavior. We now explain the rationale behind the definition of each theme and its sub-themes.

The Emotion theme aggregated responses that defined polyamory as the experience of certain types of feelings in a given context, most commonly feelings of love for more than one person at a time; for example, “loving many people” (40-year-old bisexual woman; in a non-monogamous relationship). A distinction in coding was made between responses that defined polyamory as the feeling itself and the existence of those feelings in a concrete relationship. The former were coded in the *Intrapersonal* sub-theme, with answers that focused on the inner emotion (love, attraction) toward multiple people rather than having a concrete relationship with them. The latter were included in the *Interpersonal* sub-theme, where feelings of intimacy, affection, romance, love, and compersion were mentioned in the context of a relationship between more than two people, with some participants highlighting that feelings must be felt equally for all partners; for example, “liking more than one person, in equal levels” (29-year-old heterosexual man; in a monogamous relationship). Some answers also conveyed that these feelings are innate or natural; for example, “It’s a way of living intimate relationships, in defiance of formats established by moralist and repressive societies, seeking a fuller life more in agreement of our gregarious, social nature” (42-year-old bisexual woman; in short-term relationships).

The Behavior theme encompassed responses that focused on specific behaviors among partners. The most commonly mentioned behavior was of relating to several people in some way and was included in the *Relating* sub-theme; for example, “A relationship where it is possible to love more than one person” (33-year-old woman in a monogamous relationship; undisclosed sexual orientation). Many answers qualified this relationship with feelings mentioned in the previous theme and also attributed behavioral qualifiers such as longevity, stability, and cohabitation to these polyamorous relationships; for example, “Freedom to love more than one person, building through friendship, care, respect, and love a new family or enlarging the family that existed previously” (33-year-old heterosexual woman; in a non-monogamous relationship). Some people excluded casual or short-term relationships from their definitions of polyamory; for example, “I would only call it polyamory when there’s emotional involvement with more than one person, not simply the act of having sexual relations with different partners” (29-year-old heteroflexible woman; in a non-monogamous relationship). A small number of people included casual or strictly sexual relationships in their definitions; this was more common in the few derogatory comments, where people mentioned polyamory as a means to disguise cheating or deceive someone into sex. Some people also focused on the structure of relationships and classified them as either ones that are open or closed to new partners, where people can date as individuals or where everyone involved must have a relationship with each other. Some answers included all these possibilities in the definition of polyamory and used the word as an umbrella term for different forms of non-monogamy; for example, “There’s no concrete and correct way to be polyamorous. Polyamory goes from relationship anarchy to exclusive relationships between more than two people” (37-year-old heterosexual man; in a non-monogamous relationship).

The Behavior theme also included mentions of ethics, which were grouped in the *Ethics* sub-theme. Most of these answers made mentions of either knowledge (about other partners) or explicit consent or both, but some also mentioned that partners must be honest with and respect each other (further investigation would be needed to qualify what this respect could mean). Very few mentions were made to the creation and following of rules; for example, “A relationship between at least 3 people with rules defined by all” (31-year-old heterosexual woman; in a monogamous relationship). Some participants talked about following specific ethical/moral frameworks, such as having a relationship based on feminist principles or religious ones.

The last sub-theme under Behavior was about *Sexuality* and aggregated responses that posited polyamorous relationships as being mandatorily or potentially sexual. About half of the answers coded in this sub-theme defined a polyamorous relationship as sexual in nature, whereas the other half stated that sex can happen or not, in different degrees of ambiguity; for example, “A term used for people who love more than one person independent of having sex or not” (18-year-old bisexual woman; in a monogamous relationship).

The Potential theme encompassed the codes that suggested polyamory as a possibility, something that someone is open to or capable of doing or feeling, even if not expressing these at a particular moment. This potential was mentioned in two distinct contexts, and thus divided in two sub-themes. The first one was *From without*, which includes answers coded for “Freedom to” and “Ability to” (in Portuguese *poder ter*, which can mean being capable of, but also being allowed to). This included answers that conveyed either some sort of permission or lack of external restriction for polyamory to exist; for example, “Polyamory is loving more than one person and being allowed by mutual agreement to having physical and emotional relationships with them” (22-year-old pansexual woman; in a monogamous relationship). This sub-theme suggests some participants viewed polyamory as an agreement formed within a relationship, which involves obtaining consent from a current partner to allow for the possibility of other partners. The second sub-theme was *From within,* which encompassed answers that framed this possibility as an inner capability or possibility or an openness for experience not related to external parties; for example, “The capacity of falling in love for more than one person” (32-year-old bisexual woman; in a non-monogamous relationship).

### Distinctions Between Definitions by People in Consensual Non-Monogamy Relationships and of Those in Monogamous Relationships Unwilling to Engage in Consensual Non-Monogamy

As stated above, the secondary analysis focused on differences that did not feature in the original research design. We were interested in discovering whether the in-group status concerning CNM would make any difference in how participants defined polyamory in contrast to those in monogamous relationships unwilling to be involved in CNM (out-group). We found that all the codes were present in both groups, with the exception of *longevity* and *essentialism* that were absent from definitions of polyamory presented by people involved in CNM. Furthermore, neither *longevity* nor *essentialism* was especially salient or conceptually central in responses from people involved in CNM. This means that those two topics were not fundamental in the responses put forward by that group.

People who were in CNM relationships tended to have longer answers, and these were more complex or nuanced; for example “It’s a relationship format that is non-monogamous, ethical, feminist, where there’s equal rights, with a strong family base and stable support system, with commitments that can vey from cohabitation to fuck-buddies, through non-sexual, queerplatonic relationships, where all parts have knowledge of all others and consent to this relationship format, independent of greater or smaller intimacy between themselves” (24-year-old biromantic, grey asexual woman; in a non-monogamous relationship).

People in CMN relationships focused specifically on *Interpersonal* feelings, Potential, and *Ethics*. They focused on *Interpersonal* feelings of intimacy and affection, while people in monogamous relationships focused less on those aspects, and some noted how feelings should be equal among all partners, as seen in the quote above. People in CNM relationships mentioned potentiality more in general and specifically in terms of freedom, while those in monogamous relationships mentioned a sense of being “allowed to” more often.

Conversely, people in monogamous relationships unwilling to be involved in CNM focused their responses on *Relating* behaviors, especially in the existence of a relationship; while people in CNM relationships were more specific when talking about *Relating* and mentioned commitment and cohabitation. While both groups mentioned *Sexuality*, monogamous people unwilling to be involved in CNM were more likely to state sex as a required part of a polyamorous relationship, while people in CNM relationships were more likely to describe sex as an optional part.

## Discussion

This study contributes to the literature by analyzing laypeople’s definitions of polyamory and showing the distinctions between definitions of people in CNM and of people in monogamous relationships who are unwilling to engage in monogamy. As mentioned above, there is a potential link between the usage of limited or incomplete definitions and stigma and negative impacts on people in CNM relationships (both in their personal lives and when seeking out medical or therapists’ help). Seeking out laypeople’s definitions helps to decenter academia and institutional, medical, and educational systems as the sole or main producers of knowledge about lived experiences as well as the social power associated with it.

Overall, we noted that laypeople conceptualized polyamory in terms of it being an Emotion, Behavior, and Potential. Furthermore, people in CNM relationships tended to focus their definitions on *Interpersonal* feelings, with more nuanced answers than people in monogamous relationships who were unwilling to participate in CNM, whose replies focused more on sex or on being “allowed to” have multiple relationships.

As noted previously, when referring to one possible definition, a study evaluating the general population’s understanding of polyamory suggests most people do not have a comprehensive understanding of polyamory (Rubel & Burleigh, 2020), and definitions of polyamory are varied and contested. In this study, we sought to understand what themes different respondents would mobilize to create their definitions of polyamory, rather than starting out with academically-derived or activist-derived definitions.

Most people in the sample would fit into the category of having a “basic understanding” of polyamory, as categorized in Rubel and Burleigh’s (2020) study, which leaves out the topic of consent. This is relevant, as it can point toward a lack of distinction between “cheating” in monogamous relationships and CNMs, which can affect how people perceive, and thus react to, CNM relationships. As explored above, there seems to be added moral condemnation of “cheating” in monogamous relationships in comparison to that in CNMs (Grunt-Mejer & Campbell, 2016); however, that is not always the case (Anderson, 2010). If the distinction between “cheating” and CNM is not clear to laypersons, people might, consciously or unconsciously, associate the two and negatively evaluate CNMs. Moreover, based on the issue of consent, a less nuanced definition of what CNM encompasses might also make it harder for laypeople to empathize with CNM relationships or to consider them equally valid.

Although there are some similarities between our results and Rubel and Burleigh’s (2020) literature-derived definitions, the themes that emerged from our analysis present a tripartite approach that clearly illustrates separate but connected dimensions of conceptualizing relationships.

In our study, laypeople used answers that could arguably fit the categories— stemming from academic and activist work—these authors put forward, such as identity, belief, status, and agreement. However, the current study’s participants rarely presented them using those terms, with the exception of mentions of polyamory as a relationship status. Very few people in our sample defined polyamory as an identity; the most represented meaning defined polyamory as a relating Behavior. To this, the closest analogue in their study was the aforementioned “relationship status.” A sizeable minority of participants also characterized polyamory as a possibility, which in the definitions collected by Rubel and Burleigh (2020) is closer to a set of beliefs, especially among people already involved in CNM relationships—however, a possibility more directly frames the way people conceive of a given relationship. Polyamory as a relationship agreement was also not common in our sample; however, this might be because of linguistic ambiguity in Portuguese, which suggests that in further studies, definitions based on relationship status and relationship beliefs might be more effective than those based on relationship agreements and identity.

Thus, our study shows that academic and popular-literature structuring of definitions can benefit from being critically reconceptualized from laypeople’s approach. Moreover, the study shows that laypeople from our sample conceptualize relationship dimensions differently from academic and popular literature and also value them differently. Likewise, when addressing relationship structures, academics, educators, and health providers should consider how people who relate to CNM in different ways can perceive their own (and others’) experiences in disparate ways and prioritize different dimensions of their relationships.

Furthermore, in contrast to the specialist definitions collected by Rubel and Burleigh (2020) *in toto*, the definitions given by our respondents demonstrate a lot more nuance. The following reasons clarify this argument: (1) Besides distinguishing between romance and intimacy, respondents also brought up affection, love, and compersion; (2) cohabitation is shown to coexist with, but be separate from, relationship structure and duration, meaning that typical hallmarks of committed relationships were put into question when laypeople from our sample defined polyamory; (3) the ethical dimension goes beyond consent, incorporating other elements such as knowledge, respect, honesty, and political and moral frameworks like feminism; (4) there is an element of recognition of potential conflict when polyamory as a potential is framed in light of external constraints acting upon the polyamorous subject (be it from relationship dynamics, e.g., preexisting hierarchies impinging on relationships, or from mononormative contexts, e.g., when people have to cope with professional or personal discrimination).

Familiarity with the concept might help explain some of these results as well as the sample’s characteristics (younger, more educated participants vis-à-vis the general population). Even so, it would support our hypothesis that more exposure and more visibility of CNM relationships can lead to more acceptance, as stereotypes are replaced by more nuanced understandings of these relationships.

Most responses had a single definition of polyamory, while a significant minority gave broader definitions with great latitude for different relationship structures and possibilities, such as open or closed relationships, relationships based on agreements, rules or the absence of them, and sexual or platonic relationships; some even mentioned polyamory as an umbrella term for other forms of CNM relationships, such as open relationships or relationship anarchy. While people might have given shorter answers for brevity or comfort (e.g., when typing on a smartphone), within the multiple and contested definitions of polyamory, it seems most people tend to adhere to just one; here, we observed a tendency for people in CNM relationships to have a broader understanding of possible multiple layers of definition than the average population.

Overall, these definitions paint a complex picture of what polyamory represents, reinforcing the contested nature of its definition (Kean, 2018; Klesse, 2005, 2006). This complexity comes from the three abovementioned dimensions (Emotion, Behavior, and Potential) and the detail in which they go, which is often absent from other more widely circulated definitions. It also shows that polyamory cannot be seen to be representing a specific thing but rather a constellation of different approaches to Behavior, Emotion, and Potential.

Since there were some observable differences between the in-group and out-group, we posit that some of this added complexity and nuance (more common in the in-group) can be related to personal experiences and attitudes around emotions and intimacy and personal experiences, attitudes, and/or literacy about CNM. It might also reflect access to, or participation in, cultural shifts like “designer relationships” (Michaels & Johnson, 2015), where relationships are seen as a blank slate meant to be co-created by those involved in any type of relationship, thus attempting to counterpoint mononormativity. Likewise, as we will explore below, different facets of polyamory and how it is understood can be strategically deployed to counteract and reinforce stigma and social acceptance.

This also shows that when the naturalness of monogamy is contested, these dimensions become more apparent and are more problematized. If we argue that experience and literacy can be fundamental in shaping definitions and understandings of polyamory (and other CNMs), relationship literacy (Trahan, 2014) and positive media representations become paramount to counter the stigma against CNM (Cardoso, 2020; Town, 2020), since monogamous people are less likely to have that experience (even indirectly through friends and family) or literacy.

### Stigma

Our study shows that people in monogamous relationships unwilling to be involved in CNM see polyamory as sexual, more so than their non-monogamous counterparts. Furthermore, they focus less on interpersonal feelings, especially intimacy and affection; this means, they see polyamory as more instrumental and less embedded in meaningful relationships. This might be one way that stigma appears in our sample and may be explained by social identity theory, more precisely by intergroup conflict (Tajfel & Turner, 1979). According to this approach, people do not identify with out-groups and may discriminate against them to promote intragroup cohesiveness, cooperation, and positive attachment. In this study, our definition of out-group refers to monogamous people who are unwilling to be involved in CNM. For this group, it may be the case that polyamory is defined by specific characteristics that are less favorable for monogamous people to promote in-group favoritism, that is, group members favor their group to the detriment of other groups. This possibility is in line with the results found by Sizemore and Olmstead (2018) with emerging adults, who found that participants unwilling to get involved in CNM presented a more mononormative approach to relationships, that is, the idea that monogamous relationships are better than the others and “CNM was, by default, less serious, only about sex, unsafe, less loving, less romantic, and less committed, and that such relationships were less meaningful” (Sizemore & Omstead, 2018, p. 1428).

This reinforces the idea that monogamous people contribute to stigma about CNM and that this is the outcome of their own intergroup experiences and of how their social identity is reinforced by considering other groups as less valuable. Thus, the lack of direct and indirect experience with CNM as well as identarian reinforcement might be the reason for formulation of more simple definitions given in our sample by people in monogamous relationships unwilling to engage in CNM; this further affects the understanding regarding stigma against CNM people and relationships.

While there were few derogatory comments in which polyamory was framed as unacceptable, they mostly depicted polyamory as just sexual and not true love, resulting in its status as “lesser than” and as one that originated from the out-group. This is in line with existing qualitative research with laypeople on stigma about polyamory, with what we here term in-group participants contesting definitions of polyamory that connect it to sexuality to avoid stigma, especially regarding promiscuity (Kean, 2018; Klesse, 2005, 2006).

There is some evidence that people in CNM relationships are subject to dehumanization, that is, people do not attribute human specific emotions and behaviors to them. This can be glimpsed in our sample, as monogamous people unwilling to engage in CNM mentioned *Intrapersonal* feelings, such as love and intimacy, with negative connotations (i.e., the *lack* of love as definitional). This lends weight to the hypothesis that people who are not in CNM relationships tend to acknowledge less of these characteristics in polyamorous relationships than the people who live them; these findings agree with the results in Rodrigues et al. (2018). Another concurrent interpretation is that the out-group tends to ontologize romantic love or intimacy in ways that explicitly preclude the inclusion of polyamorous relationships (Cardoso & Ribeiro, 2016), thus not incorporating these attributes in their conception of polyamory.

This perception of polyamory as “lesser than” might contribute to polyamory being stigmatized and polyamorous individuals being subject to minority stress, even when polyamory is concealed. This has been corroborated by studies on this specific aspect (Conley et al., 2012, 2013; Séguin, 2019).

Many people defined polyamory as a concrete relationship or concurrent feelings for multiple people while not mentioning consent; this agrees with the results reported by Rubel and Burleigh (2020). While the limitations of our study make it impossible to clarify this, these people defined polyamory as having multiple partners while maintaining a monogamous agreement, thereby equating polyamory to infidelity or cheating or viewing it as an excuse or justification to engage in unethical behavior. Such a view adds to social stigma associated with CNM, conflating polyamory and cheating and ignoring the importance polyamorous people place on consent and responsibility (Perez & Palma, 2018).

Conversely, people in CNM relationships in our sample tended to include more detailed understandings of dynamics that surround consent (e.g., knowledge and respect). This lends strength to the idea that it is important to socially and experientially situate those who produce knowledge about lived experiences (Haraway, 1988). Moreover, it supports the idea that academics and scholars should pay close attention to opinions of polyamorous people when constructing their definitions. This study contributes toward this goal by showing the many different dimensions laypeople use to conceptualize polyamory. In addition, the study shows that their definitions can and should serve as the basis for academic work.

Lack of attention to polyamorous people’s lived experiences might unwittingly contribute toward promoting stigma by occluding features and dimensions that polyamorous people consider fundamental. This does not mean that out-group definitions should be discarded, as they can serve as an indicator of lacunae in literacy, visibility, and experience of a person, which might help inform knowledge production and empirically-supported health and education initiatives, some of which have already been flagged (Davidson, 2002). We note that significant contributions have been made in this aspect by altogether replacing the theoretical frameworks used to address these issues, namely through the concepts of “gender, sexual and relationship diversity (GSRD)’” by Barker (2017) and “Sexual Configurations Theory” by van Anders (2015), which reconceptualize how we address intimacy and sexuality by not departing from a normative or central position, from which “Other” positions would be constituted.

People who suffer stigma have to face the task of managing social expectations. Although polyamorous relationships are often closeted, even concealed stigma can lead to psychological and physiological health implications (Conley et al., 2012, 2013). Our data shows people in CNM relationships tend to present definitions that seem shaped as if trying to avoid sexual stigma and embrace social norms by minimizing the salience of the sexual dimension of polyamory. Additionally, they also appear to be trying to assert a social and political critique on monogamy as a system, offering new ways to express affectivity and develop relationships out of normativity’s bounds, expressing the will for a politically engaged approach to relating (Cardoso, 2014, 2015).

Simultaneously, attempts at inclusivity of asexual polyamorous people can also be misconstrued as attempts at downplaying the role of sex in polyamorous relationships (Scherrer, 2010). Since romantic discourse is usually sex-normative (i.e., it assumes that sex is part of a romantic relationship), questioning a normative dimension of sexual and intimate norms might unwittingly reinforce others.

### Limitations

In this study, a convenience sample was used and, therefore, is not a complete representative of the Portuguese population. As such, some sampling biases are present, namely, the snowball sampling starting from the researchers’ social networks might be connected to higher levels of familiarity with the concept. However, it should be noted that at the time of the survey, most of the sample population was not in a CNM relationship and that a significant number was, furthermore, unwilling to be in CNM relationships.

Due to the characteristics of the overall project from where these results originate (pertaining to statistical analysis), the survey only encompassed people who identified as men or women (regardless of them being cisgender or trans).

The people in our sample also had a higher rate of high-education status than the Portuguese average (52% vs. 15%, according to the National Statistics Institute [INE, 2016]) and were younger than the Portuguese average population (32.19 vs. 41.83, according to INE, 2013). Moreover, the sample likely included a higher representation of LGBTQ people. Till date, no national study has been conducted to verify the demographic distribution of sexual orientation and/or identities, but in other western countries, the LGBTQ population varies in the single digits: 3.4% in the U.S. (Gates & Newport, 2012) and 2.5% in the UK (Geary et al., 2018). In our sample, over 30% of participants identified as LGB. Some studies on the U.S. population show that although non-monogamous behavior has no association with education, it does have an association with sexual orientation, with LGB people being more likely to have had a sexually non-exclusive relationship (Haupert et al., 2017; Organisation for Economic Co-operation and Development [OECD], 2015). To the best of our knowledge, although the prevalence of non-monogamous relationships in Portugal has not ever been measured in a representative sample, and thus no comparisons can be made, this sample has a significant number of people currently in non-monogamous relationships. In this study, the answers given were mostly short, and the survey methodology does not allow for follow-up questioning and ambiguity clarification, since there was no interaction with us.

The use of “consensual non-monogamy” is not without conceptual problems, as it still marks monogamy as the default assumption; however, it has been used as an umbrella term under which many communities recognize themselves. Therefore, in the present context, we considered it to be the least-bad term to use and preferable to using a term that draws no self-recognition from large swathes of the population we are addressing, even though it may ironically contribute to maintaining the centrality of monogamy.

There is no way to guarantee that participants did not research definitions online while answering the survey. Thematic analysis is a qualitative research methodology. This methodology is based on a constructionist epistemology, as language and meanings are constructed and contested by those who use them. Therefore, even conflicting or contradictory themes can be generated. We strived for a bottom-up generation of themes, giving precedence to terms and meanings present in the sample. Moreover, as Braun and Clarke remind us, and in line with Taylor and Ussher’s argument, there is an “*active* role the researcher always plays in identifying patterns/themes, selecting which are of interest, and reporting them to the readers” (cited in Braun & Clarke, 2006, p. 7, emphasis in the original). We have studied CNMs and polyamory in the past and are familiar with current literature on the topic. As such, some of the themes might be influenced by each of our prior research interests.

### Future Studies

Considering the study’s results and limits we consider that it is important to further investigate and deepen this area of inquiry. In our opinion, future study could depart from a social categorization perspective by considering people who identify and do not identify with CNM as well as their experience with CNM. This would help understand how current and past experiences with CNM shape people’s understanding of it. Furthermore, such studies need to be replicated in different cultural contexts, to better understand and capture the sociocultural and historical variance and situatedness of how certain descriptors are appropriated by different cultures and how ideas travel, both physically and temporally.

Furthermore, the layperson’s definitions of polyamory should also be collected in different cultures and countries, to establish potential cultural differences between how the seemingly same relationship structure or identification is perceived and how that connects to the lived experiences of it, as well as with experiences of discrimination. To overcome some of the limitations described in this study, criteria for in-group and out-group should be established a priori, and different forms of conceptualizing in-group and out-group status should be deployed to better understand the best approach to differentiate between the two.

Given the aforementioned limitation regarding gender, encompassing a greater diversity of gendered experiences and analyzing responses as they connect to those gendered experiences will also be important. This will allow future studies to understand how discrimination operates differently for people who are socially disadvantaged in terms of their gender identity.

In addition, studies should approach stigma and stereotype reduction techniques and interventions experimentally, such as with exposure to inclusive media, education programs, and awareness-raising training for professionals and educators.

### Implications

Understanding laypeople’s definitions of polyamory can contribute to a better understanding of perceived stigma and new strategies to avoid it. The definitions presented in this study can also orient definitions used in other studies, especially considering sex as a possibility instead of a central/defining characteristic of polyamorous relationships. Moreover, future studies with relationship structures that deviate from mononormativity can help understand such relationships and the way monogamy operates as a social system; the studies can also help understand underlying assumptions by offering an explicitly contrasting perspective and positioning some traits usually associated only with monogamy as being also present in other relationship types.

This study shows how polyamorous relationships can be seen as offering a wide range of intrapersonal and interpersonal emotions that are valued by the participants in a variety of ways, rather than always adhering to stereotypes held by monogamous people, such as the existence of a mandatory sexual aspect to polyamorous relationships, prevalence of the exact same emotions for all involved, or considering “designer relationships” as not possible or as inferior.

In our sample, monogamous people unwilling to engage in CNM often neglected aspects in their definitions that seemed important to participants in CNM relationships, such as informed consent, intimacy, cohabitation, and building families. Health professionals, educators, social workers, and other stakeholders who directly impact people’s lives in a professional or institutional capacity should be aware of these distinctions between how polyamory is presented by people in monogamous relationships or who identify themselves as monogamous and by those in CNM relationships or who self-identify to have interest in non-monogamies; this would serve as a way to not further stigmatize their non-monogamous patients and prompt monogamous people in different contexts (e.g., health services, community services) to question their assumptions and relationship dynamics. This includes making sure that they themselves do not internalize or project these stereotypical assumptions in clinical settings.

Academic debate understands polyamory as a contested term, thus reflecting the perceptions of laypeople, who also tend to not have a single definition of polyamory. When considering relationship diversity and relationship orientation, it is important to acknowledge that many modes of expressing this form of non-monogamy exist and that they encompass a broad range of behaviors and identities. This is important for clinicians, activists, policy makers, and others involved in situations that might impact public perceptions or experiences of intimate relationships.

Through our contribution, we hope to advance the debate on how to define polyamory and to bring awareness to often-relegated categories of experience that might, in fact, be central to the lived experiences of many polyamorous people. Furthermore, we hope to demonstrate that relying on only academic and specialist definitions might actually hinder research, education, and health intervention. By looking at laypeople’s definitions, we hope to have opened up a wider array of variables and salient elements that can be incorporated into discussions of relating, regardless of context. Additionally, we have illuminated how the conflating of theoretical cognates (e.g., love with intimacy or with romance) might be counterintuitive for many. Through the deconstruction of assumptions around what relating means (as it pertains not only to polyamory but also to any other relationship configuration), we hope to also facilitate the creation of better teaching and therapeutic resources, since more nuanced facets of people’s lived experiences can be explored autonomously.

## Footnotes

1 All theme names are presented with the name of the theme capitalized and sub-theme names are presented in italics.
