# Correction to: Defining Polyamory: A Thematic Analysis of Lay People’s Definitions

**DOI:** 10.1007/s10508-021-02113-6

**Published:** 2021-07-30

**Authors:** Daniel Cardoso, Patricia M. Pascoal, Francisco Hertel Maiochi

**Affiliations:** 1 Department of Sociology, Manchester Metropolitan University, Manchester, M15 6EB UK; 2 Escola de Comunicação, Artes E Tecnologias, Universidade Lusófona de Humanidades E Tecnologias, Lisboa, Portugal; 3 CICPSI, Faculdade de Psicologia, Universidade de Lisboa, Lisboa, Portugal; 4 Escola de Psicologia E Ciências da Vida, Universidade Lusófona de Humanidades E Tecnologias, Lisboa, Portugal; 5 CPUP, Faculdade de Psicologia E, Ciências da Educação da Universidade Do Porto, Porto, Portugal

## Correction to: Archives of Sexual Behavior (2021) 50:1239–1252 https://doi.org/10.1007/s10508-021-02002-y

The article “Defining Polyamory: A Thematic Analysis of Lay People’s Definitions”, written by Daniel Cardoso, Patricia M. Pascoal, and Francisco Hertel Maiochi, was originally published electronically on the publisher’s internet portal on 27 May 2021 without open access. With the author(s)’ decision to opt for Open Choice, the copyright of the article changed on 20 July 2021 to © The Author(s) 2021 and the article is forthwith distributed under a Creative Commons Attribution 4.0 International License, which permits use, sharing, adaptation, distribution and reproduction in any medium or format, as long as you give appropriate credit to the original author(s) and the source, provide a link to the Creative Commons license, and indicate if changes were made. The images or other third party material in this article are included in the article's Creative Commons license, unless indicated otherwise in a credit line to the material. If material is not included in the article's Creative Commons license and your intended use is not permitted by statutory regulation or exceeds the permitted use, you will need to obtain permission directly from the copyright holder. To view a copy of this license, visit http://creativecommons.org/licenses/by/4.0/.

